# High acceptability for cell phone text messages to improve communication of laboratory results with HIV-infected patients in rural Uganda: a cross-sectional survey study

**DOI:** 10.1186/1472-6947-12-56

**Published:** 2012-06-21

**Authors:** Mark J Siedner, Jessica E Haberer, Mwebesa Bosco Bwana, Norma C Ware, David R Bangsberg

**Affiliations:** 1 Department of Infectious Disease, Massachusetts General Hospital, Harvard Medical School, 55 Fruit St., GRJ-5, Boston, MA, 02114, USA; 2 Harvard Medical School, Boston, MA, USA; 3 Mbarara University of Science and Technology, Mbarara, Uganda; 4 Ragon Institute of MGH, MIT and Harvard, Massachusetts General Hospital, Center for Global Health, Boston, MA, USA

**Keywords:** SMS, Cellular phones, HIV, Sub-Saharan Africa, Confidentiality, Privacy

## Abstract

**Background:**

Patient-provider communication is a major challenge in resource-limited settings with large catchment areas. Though mobile phone usership increased 20-fold in Africa over the past decade, little is known about acceptability of, perceptions about disclosure and confidentiality, and preferences for cell phone communication of health information in the region.

**Methods:**

We performed structured interviews of fifty patients at the Immune Suppression Syndrome clinic in Mbarara, Uganda to assess four domains of health-related communication: a) cell phone use practices and literacy, b) preferences for laboratory results communication, c) privacy and confidentiality, and d) acceptability of and preferences for text messaging to notify patients of abnormal test results.

**Results:**

Participants had a median of 38 years, were 56% female, and were residents of a large catchment area throughout southwestern Uganda. All participants expressed interest in a service to receive information about laboratory results by cell phone text message, stating benefits of increased awareness of their health and decreased transportation costs. Ninety percent reported that they would not be concerned for unintended disclosure. A minority additionally expressed concerns about difficulty interpreting messages, discouragement upon learning bad news, and technical issues. Though all respondents expressed interest in password protection of messages, there was also a strong desire for direct messages to limit misinterpretation of information.

**Conclusions:**

Cell phone text messaging for communication of abnormal laboratory results is highly acceptable in this cohort of HIV-infected patients in rural Uganda. The feasibility of text messaging, including an optimal balance between privacy and comprehension, should be further studied.

## Background

Transportation to clinic visits and communication between patients and providers are among the challenges that complicate optimal health care delivery in rural, resource-limited settings (RLS) [1-4]. Abnormal laboratory results are a particular challenge. When ordering tests with potentially abnormal results, clinicians and/or patients must decide between an early return visit within days to weeks, often at significant cost to the patient, or the standard months-long return with the threat of delaying a response to an abnormal result. More efficient and effective provider-patient communication strategies could improve management of abnormal laboratory results, as well as other aspects of HIV/AIDS care in RLS as noted by recent United Nations recommendations [5].

The widespread availability of mobile communication, along with its ease of use and relatively low cost make it a promising medium to improve health related communications in resource poor settings [6]. Mobile phone subscriptions have increased over six-fold globally to nearly 90 per 100 people during the period 2000–2011 [7]. The most substantial increases in cell phone access have occurred in sub-Saharan Africa, where cell phone connectivity increased from approximately 5 to 70% and subscribership increased from 16 to 380 million users from 2000 to 2008 [8]. In Uganda, access increased nearly 80-fold to 38 subscribers per 100 inhabitants over the period 2000 to 2011 [7]. Studies in resource rich areas have demonstrated efficacy of SMS text messaging to motivate an array of positive health behaviors including increased sunscreen use [9], smoking cessation [10], returning to care for sexually transmitted infection treatment [11], improved glucose control in diabetics [12,13], and weight loss in obese patients [14]. Though more studies are needed, early studies of cell phone use to improve HIV-related communication in resource limited settings have shown benefits in reducing missed clinic visits and improving medication adherence [15-17].

Despite these positive findings, few data exist on the acceptability and feasibility of cell phone text messaging for health communications in RLS [18-20]. Patient privacy, confidentiality, and literacy are important patient-centered issues to consider prior to implementing and disseminating mobile technology for health care purposes in order to promote uptake and effectiveness. At the Immune Suppression Syndrome (ISS) Clinic in Mbarara, Uganda, clinic staff has been interested in investigating cell phone communication as a means of informing patients about abnormal test results and motivate early return to clinic when appropriate. We were unable to find any prior studies about cellular phone messaging to alert patients about abnormal test results in HIV-infected populations in sub-Saharan Africa. As such, we performed a mixed-methods study of quantitative and qualitative interviews to determine: a) cell phone use practices and literacy, b) preferences for laboratory result communication, c) privacy and confidentiality, and d) acceptability of and preferences for text messaging of health information.

## Methods

### Overview

We performed a cross-sectional survey of participants recruited from the pre-visit triage room at the Immune Suppression Syndrome (ISS) clinic at Mbarara Regional Referral Hospital. The Mbarara district has a population of approximately 400,000 residents, of whom approximately 80% live in outlying rural areas [21]. Inclusion criteria for the study included age greater than 17 years and self-reported access to a cell phone. We interviewed consecutive patients presenting to the clinic who met the inclusion criteria and agreed to participate after written informed consent. A trained research assistant conducted the interview in Runyankole, the local language, using a voice recorder in a private room over 30–60 minutes after the clinical encounter. Responses were back-translated into English and entered into an online REDCap electronic database [22]. A study coordinator reviewed data entry for all 50 surveys for quality assurance.

Data were extracted and analyzed using Stata version 11.2 (StataCorp, College Station, TX). The study protocol was approved by the ethical review committees at Partners Healthcare, the Mbarara University of Science and Technology, and the Ugandan National Council of Science and Technology.

### Survey characteristics

We designed the interview to cover four primary domains of health-related communication in our patient population: a) cell phone use practices and literacy, b) satisfaction with and preferences for clinic laboratory result communications, c) privacy and confidentiality issues about health-related communication, and d) acceptability of and preferences for cell phone text messaging to notify patients about abnormal test results. We probed in detail about preferences for text message communications including preference for coded versus direct messages, optimal scheduling and content of messages, and methods to enhance patient privacy. Surveys were written in English, translated into Runyankole and then back translated into English by a separate translator to optimize fidelity of the original question format. To elicit feedback about question comprehension in the local language prior to study initiation, the survey was piloted with two local staff.

### Analyses

We determined the frequency and distribution of demographic characteristics and responses to quantitative questions. We used the Z-test of proportions to compare responses to dichotomous quantitative questions. For open-ended questions, study investigators divided responses into major themes. The complete script of the English version of the survey is available with this article (Additional file 1: Appendix 1).

## Results

### Participants

Fifty patients were enrolled and completed the survey. Four participants were not enrolled due to: lack of cell phone ownership (2), declined consent (1), or did not have the required time available to complete the interview (1). The median age of participants was 38 years (range 20–53) and 56% were female. Twenty-two participants (44%) were from the Mbarara district. The remaining 28 participants were from a total of 10 other districts in Uganda.

### Cell phone use practices and literacy

All participants reported cell-phone ownership. A sizeable minority (18%) used multiple cell phones in the past seven days, but most (80%) reported multiple cell phones in their household. Nearly half of participants (48%) use more than one cell phone carrier by way of owning multiple subscriber identity module (SIM) cards.

Most participants reported access to cell phones 24-hours per day (70%). The most common period without access was after sunset (26% of participants), when nearly one third of participants (32%) shut off their phones. All respondents reported access to their phones seven days per week.

Ninety and 44% of participants reported the ability to read and write Runyankole and English, respectively. Most participants (96%) receive and know how to open and access (90%) SMS messages. Notably, when asked, “Which of these methods are acceptable ways for your doctor to tell you about abnormal laboratory test results?” the three participants who reported they could not read Runyankole also reported acceptability of an SMS text messaging system for reporting of laboratory results. When asked specifically about their thoughts on SMS communication (“Imagine that your doctor wants you to know an important piece of information like an abnormal lab test. How would you feel if he sent you that information by SMS text message to your cell phone?”) These participants cited reliance on friends for family members for interpretation of results, for example:

"I would be very glad because I have broken the ignorance. This is a quick and a comforting way to talk to us and a clear sign that they care for us. If I get the message I call my treatment supporter to read for me the message."

Participants receive a median of 3–5 messages per week (range 0 – greater than 20). Most participants reported that they could receive SMS texts free of charge (87.5%, 29/35). In contrast, all participants (27/27) reported they were charged to send SMS messages. The median cost per message was $0.04/message (range $0.02 – 0.20).

### Satisfaction with clinic laboratory result communications

Ninety percent of participants reported they usually learn about test results from their clinician at the following visit, while an additional 8% report they do not typically find out their results. While 37 of 50 (74%) participants reported that learning the laboratory result from the physician during the following visit was acceptable, 90%, 98% and 100% also found recorded voice message, phone calls, and text messages acceptable respectively (p < 0.001 for all comparisons versus learning from physician). Though 82% reported they would return to clinic earlier if they were aware of abnormal test results, participants also reported returning to clinic is difficult because of transport costs (84%), personal illness (52%), work (18%) or family obligations (10%).

### Privacy and confidentiality issues related to health communication

Ninety eight percent of participants had disclosed their diagnosis to at least one person and 78% (32/41) had disclosed to their primary partner. Although 90% reported they were not afraid that a cell phone message would disclose their HIV-status:four participants (8%) specifically listed a fear of breach of privacy related to cell phone text messaging about health:

"I don't see any worries because even the people that use my phone they have that use it with my consent and when am there seeing them."

"I don't have any worries because this is aimed at helping my health. I don't mind privacy. I have overcome stigma and am happy this way;"

"The problem might come to couples who have not disclosed to each other this might cause a conflict if the other partner sees the message."

"We have many friends who can easily pick your phone, check your inbox, and get to know your private information. It would be good to delete the SMS after reading."

When asked what the clinic could do to ensure confidentiality of the cell phone communication system, answers typically fell into one of four major themes:

i. Text message privacy is the responsibility of the patient (13 respondents):

For those who haven't disclosed they can delete the SMS after reading the message. The issue of privacy is largely the responsibility of the receiver;

ii. Clinic staff should maintain a reliable and secure database system (12 respondents):

You should delete/protect the database from where the messages are being sent from your system…

iii. Clinic staff should provide instruction on use of the system and risks of disclosure (13 respondents):

First ask the patient on his phone usage and then you agree on how it works. Explain to the patient the possible risks so that they are aware.

iv. Ensure anonymous messages (3 respondents).

When asked further about the use of a personal identification number system to protect health-related messages (“What if we required a password that only you would know that would be required to open the message? Would this help to make your results confidential or would this inconvenience you?”), 88% (44/50) responded positively. The remaining cited challenges about remembering the PIN and unnecessary inconvenience because they had disclosed to family members and therefore declined the need for increased privacy measures.

### Acceptance of and preferences for cell phone text message communications

All participants reported that they would like to receive health-related communications via cell phone messaging and that an automated system of cell phone text messaging with information about test results at the clinic would be helpful. When asked about the benefits and risk of such a system, the majority 88% (44/50) discussed benefits of improved communication and overall clinical care:

"We at times come and if you don't ask the doctor he normally forgets to tell you but with an SMS it will help you know. At times your next visit might be a public holiday and you find the hospital closed."

"Most of us are dying out of ignorance so with a message we will quickly discover our status."

Others 12% (6/50) reported that such a system would decrease the cost of care:

"It will save the cost of transport because we come and we find nothing ready for almost four times of coming to the clinic and going with no achievement. So it would make us come when we are sure."

Though all listed benefits, a minority also expressed concerns about the system including disclosure 14% (7/50), illiteracy 8% (4/50), potential to increase stress 6% (3/50), technical issues 6% (3/50), and difficulty accessing transport 4% (2/50).

When asked to explain how they would feel personally about receiving a message on their phone with information about an abnormal test result, the majority (72%; 36/50) responded positively without reservation.

"It’s okay, because it’s quick and cost effective. It’s a better way to communicate when something is so urgent."

"I would feel fine by that because we normally come for the results after 3 months… I think this would be better to get us informed."

"This influences you to come to the clinic for consultation. This is a good way to communicate because it helps me know my status."

Some participants (26%; 13/50) expressed anxiety about learning about abnormal test values over the phone:

"It might scare you a bit but I would appreciate since he cares about me. I think this would help patients relate better with doctors, and I would act accordingly."

"It might scare me a little bit, I would like to know what else I can do, so I would come back to the clinic. I would tell a friend to help me relieve my mind…"

When asked, “Please rank from most important to least important to you the features of a text message about test results”, participants ranked i) specificity of message, ii) language, iii) privacy, iv) clarity, and v) length. Notwithstanding a preference for specificity, 24 participants (48%) prefer a direct to a pre-specified coded message (example for direct message: “Your laboratory tests are ready, please return to clinic” example of pre-specified, coded message: “ABCDEFG”).

When asked what worries they have about receiving messages, most (66%; 33/50) reported they had no worries:

"I don't have any worries because this is aimed at helping my health. I don't mind privacy. I have overcome stigma and am happy this way."

Three participants said they could be discouraged or startled by the result:

"It depends on the message if it is too bad that the sickness is beyond the doctor’s control. Otherwise I don't think this affects privacy since everyone knows my status and in case I don't understand the message then I can inquire."

Technical issues were a cause of worry for some participants (14%; 7/50):

"The only worry is when my battery is down and the message is urgent."

All participants favored a chance to respond to health communications with an opportunity to reply by SMS 84% (42/50), call back 50/50 (100%), and/or return to clinic 21/50 (42%).

When asked to describe a preferred message in their own words, 49 of 50 (98%) participants gave an example using words (at least one of “test”, “result”, “CD4”, “blood”, “treatment” or “clinic”), which could potentially reveal health-related information:

"I would like it short and clear. From the lab tests we took the results were not good come back to the clinic on this date."

Seven patients also specifically included a patient’s name in their example message:

"Mr X, from our lab tests you had abnormal results, but there is help"

The most preferred messages from a list of 8 pre-specified options were: i) “This is an important message from your doctor. You had an abnormal test result. You should return to clinic as soon as possible,”; ii) “You had an abnormal test result. You should return to clinic as soon as possible,” and iii) “Your doctor would like to talk to you. Please come to clinic.”

## Discussion

In a survey of 50 HIV-infected patients in care in rural Uganda with access to cell phones, we found nearly universal acceptability for communication of information about laboratory test results via cell phone text messages. Though most participants interviewed reported literacy with the local language, those who were illiterate also expressed interest in receiving SMS messages, stating they could identify a literate person to communicate the messages. A significant proportion of patients preferred SMS communication of laboratory results to the existing system of learning laboratory results at the next clinic visit. Participants also cited secondary benefits from cellular phone messaging of laboratory result information including improved relationship with clinic staff and providers and decreased transportation costs from less frequent clinic visits.

In this cohort, concerns about breach of privacy from cell phone messaging of health information were rare. Perceptions of and expectations for maintaining confidentiality vary by region and culture. Published literature contains little guidance on these variations. One report from the Kwazulu region of South Africa described a patient-driven practice of “shared confidentiality,” or acceptability of disclosure by providers of HIV-status to close relatives [23]. For those participants in our study who did express concerns about confidentiality, there was interest in use of both coded messages and PIN codes to protect confidentiality. Prior studies have reported some difficulty in comprehension with PIN activation [24]. There is a near complete lack of published data and great need to evaluate the acceptability and feasibility of methods to optimize the confidentiality of mobile phone communications including password protected messages, coded messages, direct voice messages, and interactive-voice response formats. We plan to compare comprehension and acceptability of coded messages and use of a PIN code as part of a planned health-communication intervention at the HIV clinic in Mbarara in future studies. Finally, while concerns about disclosure were uncommon, disclosure can cause significant social and physical harm. As such, it is important that patients understand and accept these risks prior to sending sensitive SMS information.

Another concern expressed by study participants was the potential for anxiety caused by receipt of bad news with electronic messages. All participants expressed interest in the opportunity to call back after receipt of a message and most also expressed interest in a texting option. Other strategies to mitigate anxiety from receipt of bad news include enabling walk-in appointments for patients after abnormal test results and using personal voice calls instead of messages for particularly critical results. The strategies should be considered in future use of technologies to communicate health information with patients.

Access to multiple cell phones in the home (80% of participants) and SIM cards (48%) was common in this cohort. Developers of cellular phone messaging applications for health care communication should leverage these features to increase uptake and usability of these platforms. Though patients are typically asked for a single phone number, a preferable system might allow inclusion of multiple cellular phone numbers in order of preference. This strategy might increase rates of successful patient communication by overcoming challenges related to network reliability, phone battery charge, and shared phones. One resulting challenge will be restricting information to the intended recipient. PIN code access to messages and/or coding messages, which were acceptable in this cohort, might provide a solution if it proves feasible in practice.

Our findings are similar to others who have queried patient acceptance of cell phone communications in resource poor settings. A survey of 300 patients in Durban found nearly universal acceptance (96%) for text message communications from HIV providers [18]. Another survey of approximately 30 HIV-infected patients in Peru found relatively less interest, but that a substantial a majority of HIV-infected patients (74%) were interested in receiving cell phone text message reminders about HIV medication use [19]. In contrast to our findings, a study of students in secondary school also conducted in Mbarara found that only about half of all students, and 60% of those who owned cell phones reported interest in using text messages to learn about HIV [25]. An important difference between our study population and theirs is that they interviewed perceivably healthy adolescents about preventative health information as opposed to patients in care who are seeking critical medical information about their health status.

There are several potential limitations to our study. The sample was small in order to collect detailed qualitative data. As such the quantitative estimates are imprecise. We studied a systematic sample of consecutive patients at a sub-Saharan, university-affiliated African HIV treatment center serving a rural population, and the results might not be generalizable to urban or exclusively rural settings. That said, our reported rates of disclosure to at least one contact (>90%) and to a primary partner (>75%) are similar to rates reported elsewhere in Uganda [26,27]. This suggests that our findings concerning privacy of health communications might be generalizable to other populations.

## Conclusions

Widespread availability and low cost of mobile phone technology makes it a promising medium to improve health related communication in resource-limited settings with large catchment areas. In Uganda, text messages cost between $0.02 – 0.04 to send per message and are free to receive. Because transport and other structural barriers make clinic return difficult for many patients, a system to prioritize return for sick patients could help optimize patient and clinical resources. Given our findings of high acceptability for cell phone text messages among HIV-infected patients at a public clinic in rural Uganda, further study of the efficacy of this medium to improve patient-provider communication should be pursued. Mechanisms to balance patient privacy with fidelity of communication will be important for implementation.

## Competing interests

The authors report that they have no competing interests.

## Authors’ contributions

MS took the lead in study design, survey composition, analysis of data, and composition of the manuscript. JH assisted with study design and survey composition as well as significant review and editing of the manuscript. MB assisted with survey design and edited the manuscript. NW assisted with survey design and edited the manuscript. DB conceived the study, assisted with survey composition, and edited the manuscript. All authors reviewed and approved of the final version of the manuscript.

## Pre-publication history

The pre-publication history for this paper can be accessed here:

http://www.biomedcentral.com/1472-6947/12/56/prepub

## Supplementary Material

Additional file 1 Cell Phone Messaging Acceptability Survey. Click here for file
