# Supplementary material for: High acceptability for cell phone text messages to improve communication of laboratory results with HIV-infected patients in rural Uganda: a cross-sectional survey study
Source: BMC Med Inform Decis Mak. 2012 Jun 21;12:56. doi: 10.1186/1472-6947-12-56 (PMC3470938; doi:10.1186/1472-6947-12-56)
Supplement: Additional file 1 — Cell Phone Messaging Acceptability Survey. [file 1472-6947-12-56-S1.pdf]

ISS ID# Study ID Acrostic	Participant Gender	Age	District/ Parish	Interviewer's initials	Date this form was completed
___ -- ___ -- ___ ___ ___ ___ ___ ___ ___	☐ Male ☐ Female	____	D___________ P___________	__ __ __	__ __/__ __/ __ __ __ __ day month year

APPENDIX 1: Cell Phone Messaging Acceptability Survey

This interview should be done with the subject in a private place. Record all questions with a digital voice recorder for later transcription.

READ: I'm now going to ask you some questions about using a cell phone to communicate laboratory test results to you. Please tell me how you actually feel about it and do not worry about what I think. We really want to know your opinion. I will record your responses, so that I may write them down accurately at a later time. I will then delete the recording to protect your confidentiality.

First I'm Going to ask you some questions about your cell phone ownership and use.

1. Do you use a cell phone? ☐Yes ☐ No — If no, end survey.

2. How many different cell phones did you use in the last 7 days to receive or make calls?
   ☐1 ☐2 ☐3 ☐More than 3

3. How many people in the household in which you reside own cell phones?
   ☐1 ☐2 ☐3 ☐More than 3

4. Do you own a cell phone? ☐Yes ☐ No — If no, skip to question 8.

   4B. What kind of cell phone? ☐ Nokia ☐ Tecno ☐ Samsung ☐ Other _________
   4C. What service company? ☐ MTN ☐ Orange ☐ Uganda Telecom ☐ Zain/Airtel ☐ Warid ☐ Other _______________
   4D. Do other people share the cell phone with you? ☐Yes ☐ No
   4E. If yes, Whom:
   Parent: ☐Yes ☐ No
   Child: ☐Yes ☐ No
   Sibling: ☐Yes ☐ No
   Partner: ☐Yes ☐ No
   Neighbor: ☐Yes ☐ No

5. Do you turn your cell phone off at night? ☐Yes ☐ No

6. How do you charge your cell phone?

☐ electricity at home ☐ roadside stand ☐ solar charger ☐ other _________

7. How often does your cell phone battery run out?
☐Everyday ☐ Every 2-6 days ☐ Once per week ☐ Less than once per week

8. How many cell phone calls did you make yesterday?
☐None ☐1-2 ☐3-5 ☐6-10 ☐11-20 ☐>20

9. How many cell phone calls did you receive yesterday?
☐None ☐1-2 ☐3-5 ☐6-10 ☐11-20 ☐>20

10. What do you make phone calls about (tick all that apply)?
☐ conversation with friends or family ☐ practical arrangements (like when and where to meet someone) ☐ business matters ☐ other ______________________

11. Please check all of the times of the day when you have a cell phone you can use (check all boxes that are correct)?
☐Before sunrise ☐Sunrise to midday ☐Midday to sunset ☐sunset to sleep hours

12. How many days each week do you have access to a cell phone?
☐1 day ☐Some days (2-3) ☐Most days (4-6) ☐Everyday

13. Do you know how to read English? ☐Yes ☐ No
14. Do you know how to write English? ☐Yes ☐ No
15. Do you know how to read Runyankole? ☐Yes ☐ No
16. Do you know how to write Runyankole? ☐Yes ☐ No

17. When the doctor at ISS clinic orders blood tests, how have you learned about the results?
☐ Doctor tells me at my next visit
☐ I receive a phone call with results
☐ I call my doctor or the clinic to receive the results
☐ I receive an SMS text message with results
☐ I do not usually find out
☐ a family member or friend tells me
☐ Other:
____

Now I'm going to ask you some questions about sending SMS text messages.

18. Do you know how to send SMS text messages? ☐Yes ☐ No

If no, skip to question 23.

19. Do you send SMS text messages? ☐Yes ☐ No

20. About how many SMS text messages did you send in the past week?
    ☐None ☐1-2 ☐3-5 ☐6-10 ☐11-20 ☐>20

21. How much does it cost for you to send a SMS text message? ______ shillings

22. What do you send SMS about (tick all that apply)?
☐ conversation with friends or family ☐ practical arrangements (like when and where to meet someone) ☐ business maters ☐ other ______________________

Now I'm going to ask you some questions about receiving SMS text messages.

23. Do you receive SMS text messages? ☐Yes ☐ No

If no, skip to question 27.

24. Do you know how to access SMS text messages you receive? ☐Yes ☐ No

25. About how many SMS text messages did you receive in the past week?
    ☐None ☐1-2 ☐3-5 ☐6-10 ☐11-20 ☐>20

26. How much does it cost for you to receive a SMS text message? ______ shillings

Next we will ask you about your preference for learning about test results.

27. Imagine that you have a blood test with abnormal results and your doctor wants to tell about it. How would you prefer to learn about the results?
    ____
    ____
    ____

28. Which of these methods are acceptable ways for your doctor to tell you about abnormal laboratory tests (check all that apply)?

☐ Doctor tells me at my next visit
☐ I receive a recorded voice message with results
☐ I receive a phone call with results
☐ I receive an SMS text message with results
☐ Other:
____

29. Imagine that your doctor wants you to know an important piece of information like an abnormal lab test. How would you feel if he sent you that information by SMS text message to your cell phone?

[The purpose of this question is to learn what patients think about receiving medical information over their cell phone. Prompts could include: Does it seem like a good way for doctors to communicate with patients? What would you do with the information?]

30. What worries do you have about receiving a cell phone SMS text message from your doctor? *[Prompts may include: Are you worried about your privacy? Are you concerned you wouldn't understand the message?]*

31. What can we do to make sure text messages respect your privacy?

____

____

____

32. What if we required a password that only you would know that would be required to open the message? Would this help to make your results confidential or would this inconvenience you?

____

____

____

33. Would you prefer a message that is direct (READ: "Your laboratory tests are ready, please return to clinic) or coded (READ: "It is going to rain today" or "ABCDEFG")?
    ☐ Direct
    ☐ Coded

34. Please check all that apply about your feelings of receiving SMS text messages about health information on your cell phone.
    a. I would like to receive health information from my doctor by SMS text message.
    ☐Yes ☐ No
    b. I am not interested in receiving health information from my doctor by SMS text message. ☐Yes ☐ No
    c. I am afraid other people would learn about my health problems if I received SMS text messages from my doctor. ☐Yes ☐ No
    d. I think receiving SMS text messages would be a helpful way to learn about important health information. ☐Yes ☐ No
    e. I do not use a cell phone very often, so would not be able to get information this way.
    ☐Yes ☐ No

35. a. Do you think a system for sending you important health information from your doctor to your cell phone with an SMS text message would be helpful for patients in this clinic?
☐Yes ☐ No
b. Why or why not?
(*The purpose of this question is to learn what patients think are reasons why an SMS text message system to tell them about important health information would or would not work. What do they think the problems with the system would be? Prompts could include: Would there be technical problems? Would people have cell phones available to them?)*

____

____

____

____

36. What would you do if you received a cell phone SMS text message from your doctor asking you to return to clinic in 7 days because of an important health problem?
[The purpose of this question is to learn why patients might not return to clinic if their doctors asked them to with an SMS text message Prompts might include: Do you have transportation to clinic when you need it? Can you leave work or your children without planning ahead?]

____

____

____

____

37. Which of these reasons might affect your ability to return to clinic within 7 days if you received a text message informing you of a positive test result (check all that apply)?
☐Cannot get transport or afford to come back to clinic
☐Cannot miss work
☐Must take care of family members
☐Feel sick
☐Other ________________

38. If you received a confidential text message from your doctor with an important health message, your doctor may prefer that you respond to confirm that you received it. How would you feel about that?

____

____

____

39. Do you think that it would be helpful to respond and confirm that you received it?
    ☐Yes ☐ No

40. If yes, how would you like to respond?
    ☐ SMS message ☐Call Back ☐ Return to clinic
    ☐ Other (explain) ______________________

41. If you were to receive information about your health as an SMS text message on your cell phone, what time of day would you prefer to receive the message?
    ☐Before sunrise ☐Sunrise to midday ☐Midday to sunset ☐sunset to sleep hours
    ☐Anytime

42. If you were to receive information about your health as an SMS text message on your cell phone, what days would you prefer to receive the message?
    ☐Weekday (Monday – Friday, Workdays) ☐ Saturday ☐ Sunday (church day)
    ☐ Anyday / No preference

43. Have you told anyone aside from your doctor about your HIV diagnosis?
    ☐Yes ☐ No

If no, skip to question 45.

44. Whom have you told about your HIV diagnosis?
   a. Mother: ☐Yes ☐ No
   b. Father: ☐Yes ☐ No
   c. Child: ☐Yes ☐ No
   d. Sibling (brother or sister): ☐Yes ☐ No
   e. Partner/Husband or Wife: ☐Yes ☐ No
   f. Neighbor: ☐Yes ☐ No
   g. Employer: ☐Yes ☐ No
   h. Employee: ☐Yes ☐ No
   i. Workmate: ☐Yes ☐ No
   j. Religious leader: ☐Yes ☐ No
   k. Friend ☐Yes ☐ No
   l. Other ____________

45. Do you think that receiving an SMS text message would allow others to know about your HIV diagnosis?
   ☐Yes ☐ No ☐ Don't Know

46. What is an example (in your words) of an SMS text message that you would like to receive from your doctor about important health information?
   ____

   ____

47. Please rank from most important to least important to you the features of a cell phone message about test results:
   a. Privacy/Anonymity ______ (Most Important)
   b. Clarity ______ (2nd most important)
   c. Length ______ (3rd most important)
   d. Specificity ______ (4th most important)
   e. Language (English vs. Runyankole) ______ (least important)

48. Below is a list of sample SMS text messages that a doctor might send to tell you about important health information. **Please select the ones that you like** (you can select as many as you like). Here are some features of the message we would like to stress (read the following):
1. We want the message to be clear.
2. We want the message to tell you that the doctor has important information to tell you.
3. We want it to be clear that you need to return to clinic.
4. We want the message to be private. We want to be sure that if someone else reads the information, they will not know it is about you or anything about your health.

☐ a. This is an important message from your doctor. You had an abnormal test result. You should return to clinic as soon as possible.

☐ b. You had an abnormal test result. You should come to clinic as soon as possible.

☐ c. You should come to clinic as soon as possible.

☐ d. Your doctor would like to talk to you. Please come to clinic.

☐ e. Please come to clinic

☐ f. It's time for your meeting.

☐ g. Please come see me when you can.

☐ h. ABCDEFG (a non-specific code that is given at the testing appointment)

49. For the messages you selected above, please give us your opinion about the message. Tell us why you selected the message. Is it clear? Does it protect your privacy?

a. Selected Message 1 ___ (write letter a-h)

____

____

b. Selected Message 2 ___ (write letter a-h)

____

____

c. Selected Message 3 ___ (write letter a-h)

____

____

d. Selected Message 4 ___ (write letter a-h)

____

____

READ: Thank you. We are now done with the interview.
